# The Centenary of the Microscope

**Published:** 1891-07

**Authors:** 


### The Centenary of the Microscope.

It is generally thought that the invention of the microscope goes back to the close of the fifteenth century, or to be more precise, to the year 1590, when, in the city of Middleburg, in Holland, two spectacle makers, named Janssen, invented both the telescope and the microscope. This date for the invention, according to which its third centenary would arrive in 1890, does not rest on authentic documents, but is based on assertions published in 1665 by the physician, Peter Borel. He denied that Galileo, Drebbel and others deserved the credit of having invented the telescope; and in order to demonstrate that the invention of that instrument, as well as of the microscope, was due to the Janssens, produced some documents which showed that the two spectacle makers, having invented the telescope in 1590, presented a specimen of it to Prince Maurice, Stadtholder of the Netherlands, and to the Archduke Albert. Later on, however, the telescope of Prince Maurice became a microscope, in a letter of William Borelli, who declared that he had always heard in Middlebrg, his native city, that the Janssens had invented these optical instruments, and further that when he was embassador at London, in 1619, he had seen in the hands of Drebbel the identical microscope that the Janssens had presented to Prince Maurice.

Professor Govi, however, in a work which demonstrates the excellence of his judgment and his vast erudition, has collected a series of documents which not only seem to restore the merit of the invention of the microscope to Galileo, but show the various vicissitudes of the discovery itself. The first hint of the transformation of the Holland telescope into a microscope is found in a little book published in 1710 by Wodderborn, a pupil of Galileo. Speaking of the wonderful qualities of the telescope, Wodderborn adds, in praise of Galileo, that "with the instrument could be perfectly distinguished the organs of motion and sensibility of the smallest animalcule," so that the particular formation of multiplied eyes in very small animals could be perceived. This new application of the telescope by himself Galileo did not deny, though he never directly affirmed it. In the National Library at Paris is preserved a letter by Cannon Tarde, in which he speaks of visiting Galileo in Florence in 1614, when the latter was sick in bed. Notwithstanding, to Tarde Galileo gave ample explanation of a microscope then in his possession.

Whether the invention of the simple microscope be due to Janssen or Galileo, to Drebbel is due the merit of having produced at Rome, in 1624, the compound microscope. The difference between the two hardly needs explanation. The simple microscope magnifies with a single lens, or with several lenses so close together that they act like a single lens. The compound microscope has two or more lenses, separated by a convenient distance from each other, and which act separately. In 1669, Eustachio Divini constructed a collossal microscope which magnified 140 times. A little after Bonannus invented a horizontal microscope which magnified 300 times.

In the seventeenth century were laid the foundations of micrography, a science which, by the study of the minute anatomical elements and their functions, has made such progress under the name of histology, and been such a fertile cause of important discoveries. With the microscope Malpighi, by the minute examination of the tissues, confirmed the theories about them he had previously formed; Leuwenhœk discovered the globules of the blood and the structure of the nervous fibres; and Swammerdam dissected insects, of the most minute organs of which he gave descriptions still considered perfect.

In the eighteenth century, observes Henocque, but few modifications were made in the microscope. To mention all the improvements made in the instrument during our century would be tedious. During the last forty years enormous advances have been made in science by the aid of the microscope, of which the usefulness has been greatly increased by the skill with which the matter to be examined is prepared, and by the aid of photography. Micro-photography dates from 1840 only; but since that date it has had an uninterrupted series of noteworthy improvements.

Besides histology created by the microscope, by which our acquaintance with the most hidden structure of organisms is constantly increasing, bacteriology, with its rapid succession of discoveries of the highest importance, owes its existence to the microscope. Those little beings, those micro-organisms, which, by the change of the medium in which their evolution is affected, can produce so much good or so much evil, and of which it takes several millions to occupy the tenth part of half an inch in space, can now be identified according to their species, notwithstanding their changeable aspect. We can estimate the rapidity of multiplication, the number, the dimensions, and the singular manner in which by dividing themselves, or by means of a sort of buds or spores, the micro-organisms reproduce themselves.

In the examination of the inorganic world the microscope has had results not less precious. The wonderful phenomena of crystalization, the exact form of the crystals, the more precise in proportion to their minuteness, the modifying properties of the light called forth by the thinnest layer of a mineral, the interior texture of rocks, all these can be studied with a precision impossible before the invention of the improved microscope, and finally not to mention all the triumphs achieved by the instrument, it has had an application which formerly would have seemed paradoxical, since the microscope has been employed to show the particulars of the nature of the surface of the planets, particulars which have been made clear by microscopic observations of instantaneous photographs.—Ernesto Mancini, *Nuova Antologia*, *Literary Digest.*

